# Cardiomyocyte-specific overexpression of GPR22 ameliorates cardiac injury in mice with acute myocardial infarction

**DOI:** 10.1186/s12872-024-03953-5

**Published:** 2024-05-30

**Authors:** Chin-Chuan Chang, Chih-Hung Chen, Shu-Yuan Hsu, Steve Leu

**Affiliations:** 1 Department of Nuclear Medicine, Kaohsiung Medical University Hospital, Kaohsiung, 80756 Taiwan; 2 School of Medicine, College of Medicine, Kaohsiung Medical University, Kaohsiung, 80756 Taiwan; 3 Neuroscience Research Center, Kaohsiung Medical University, Kaohsiung, 80756 Taiwan; 4 Division of Hepato-Gastroenterology, Department of Internal Medicine, Kaohsiung Chang Gung Memorial Hospital, Chang Gung University College of Medicine, Kaohsiung, 83301 Taiwan; 5 Department of Anatomy, Graduate Institute of Biomedical Sciences, College of Medicine, Chang Gung University, Taoyuan, 33302 Taiwan; 6 Institute for Translational Research in Biomedicine, Kaohsiung Chang Gung Memorial Hospital, Kaohsiung, 83301 Taiwan; 7 Department of Biotechnology, College of Life Science, Kaohsiung Medical University, Kaohsiung, 80756 Taiwan

**Keywords:** GPR22, Cardiomyocyte, Acute myocardial infarction, Mouse model

## Abstract

**Background:**

The activation of G protein-coupled receptors (GPCR) signaling by external stimuli has been implicated in inducing cardiac stress and stress responses. GPR22 is an orphan GPCR expressed in brains and hearts, while its expression level is associated with cardiovascular damage in diabetes. Previous studies have suggested a protective role of GPR22 in mechanical cardiac stress, as loss of its expression increases susceptibility to heart failure post-ventricular pressure overload. However, the involvement and underlying signaling of GPR22 in cardiac stress response to ischemic stress remains unexplored.

**Methods:**

In this study, we used cultured cells and a transgenic mouse model with cardiomyocyte-specific GPR22 overexpression to investigate the impact of ischemic stress on GPR22 expression and to elucidate its role in myocardial ischemic injury. Acute myocardial infarction (AMI) was induced by left coronary artery ligation in eight-week-old male GPR22 transgenic mice, followed by histopathological and biochemical examination four weeks post-AMI induction.

**Results:**

GPR22 expression in H9C2 and RL-14 cells, two cardiomyocyte cell lines, was decreased by cobalt chloride (CoCl_2_) treatment. Similarly, reduced expression of myocardial GPR22 was observed in mice with AMI. Histopathological examinations revealed a protective effect of GPR22 overexpression in attenuating myocardial infarction in mice with AMI. Furthermore, myocardial levels of Bcl-2 and activation of PI3K-Akt signaling were downregulated by ischemic stress and upregulated by GPR22 overexpression. Conversely, the expression levels of caspase-3 and phosphorylated ERK1/2 in the infarcted myocardium were downregulated with GPR22 overexpression.

**Conclusion:**

Myocardial ischemic stress downregulates cardiac expression of GPR22, whereas overexpression of GPR22 in cardiomyocytes upregulates Akt signaling, downregulates ERK activation, and mitigates ischemia-induced myocardial injury.

**Supplementary Information:**

The online version contains supplementary material available at 10.1186/s12872-024-03953-5.

## Background

G protein-coupled receptors (GPCRs) constitute the largest and most diverse group of membrane-bound receptors, characterized by a conserved structure comprising seven transmembrane α helices, an extracellular N-terminus, and an intracellular C-terminus [1, 2]. Initiation of GPCR-mediated signal transduction occurs through the sensing of molecules from extracellular stimuli, followed by activation of intracellular signaling pathways via coupling to G proteins [2, 3]. Heterotrimeric G proteins are organized in a manner utilizing one α subunit alongside one β and γ subunits [4]. Upon binding to specific ligands, GPCRs undergo a conformational change, leading to the dissociation of Gα from Gβγ subunits [4]. Gα-subunits are classically divided into four families based on similarity: Gαi/o, Gαs, Gαq/11, and Gα12/13. Each Gα-family activates a distinct profile of effectors, such as adenylyl cyclase and phospholipase C, and then continues diverse signaling cascades [5]. Apart from adenylyl cyclase pathways, GPCR signaling is also reported to regulate the activation of mitogen-activated protein kinase (MAPK) and Akt pathways [6–8].

The role of GPCRs has been extensively studied in the cardiovascular system, where activation of these receptors exerts profound homeostatic and regulatory effects. Mutation and dysregulation of GPCRs, G proteins, and downstream effectors are found to be associated with physiological dysfunction in the cardiovascular system [9, 10]. Pharmacological therapeutics targeting GPCR modulation are widely employed in managing cardiovascular diseases, including hypertension, arrhythmias, and heart failure [10, 11]. However, most identified GPCR signaling pathways in cardiac cells are primarily involved in regulating contractility and cellular hypertrophy [12]. Although recent studies have implied the involvement of GPCR in regulating apoptosis via downstream effectors and signaling cascades, such as the Bcl-2 family, NF-κB, PI3K, MAPKs, and small GTPases [13], the role of GPCRs and their downstream signalings in ischemic heart injury remains elusive.

GPR22 is an orphan GPCR expressed in the brain and heart [14–17]. In diabetic rats, decreased expression of GPR22 was observed in the right ventricle [15]. Furthermore, loss of GPR22 increased the susceptibility to functional cardiac decompensation following transverse aortic constriction (TAC)-induced ventricular pressure overload in mice [16]. Our recent study also demonstrated that maternal fructose intake during pregnancy and lactation reduces myocardial expression of GPR22 and exacerbates cardiac remodeling after induction of ventricular pressure overload [18]. However, the role of GPR22 in myocardial ischemic stress response remains unclear. In the present study, we generated a transgenic mouse model carrying cardiomyocyte-specific GPR22 over-expression to directly investigate the role of GPR22 in ischemic myocardial infarction and elucidate its underlying mechanisms.

## Materials and methods

### Ethics

All animal experimental procedures were approved by the Institute of Animal Care and Use Committee at Kaohsiung Chang Gung Memorial Hospital (no. 2019092502). All methods are reported in accordance with ARRIVE guidelinesc(https://arriveguidelines.org) for the reporting of animal experiments [19].

### Generation of cardiomyocyte-specific GPR22 overexpression mice

For mice production, a linearized plasmid carrying myosin heavy chain 6 (MYH6) promoter and 3X flag-tagged mouse GPR22 cDNA (MYH6-Flag-GPR22) was diluted with injection buffer to a final concentration of 100 ng/µl. Four-week-old C57BL/6J female mice were super-ovulated with 3.75-5 i.u. of PMSG (Sigma-Aldrich G4877) followed by 3.75-5 i.u. of hCG (Sigma-Aldrich CG1063) 46 hours later. Super-ovulated female mice were set mating to C57BL/6J male mice, and one-cell stage zygotes were collected on the next day. Diluted MYH6-Flag-GPR22 plasmid was injected into the zygotes. Injected zygotes were cultured in KSOM in a humidified incubator (37 ^o^C, 5% CO2) overnight. 2-cell stage embryos were transferred into the oviduct of 0.5-dpc pseudo-pregnant ICR female mice. The primer set: 5’-CCCCCCGCTAGCCTAGTCTGTGACAACCTG-3’ (mGPR22-C-R-NheI) and 5’- AATTCTGCGGCCGCCATGTCAGAATTGTCAATG-3’ (mGPR22-N-NotI-F) was used for synthesizing full-length mouse GPR22 and subseqnet genotyping.

### Animals and induction of acute myocardial infarction

All animal experiments in this study were housed in an Association for Assessment and Accreditation of Laboratory Animal Care International (AAALAC)-certified animal facility in our hospital with controlled temperature and light cycles (24 °C and 12/12 light cycle). Mice were placed in a supine position on a warming pad at 37 °C after being shaved on the chest and then intubated with positive-pressure ventilation with room air using a Small Animal Ventilator (30 ml/min, SAR-830/A, CWE, Inc., USA) with inhalation of 2% isoflurane. Under sterile conditions, a left thoracotomy was performed at the level of the 5th intercostal space to expose the heart. AMI was induced by left coronary artery ligation approximately 2 mm below the left atrium using a 7 − 0 prolene suture. The induction of AMI was confirmed by the rapid discoloration observed over the anterior surface of the left ventricle, accompanied by the development of akinesia and dilatation within the at-risk area.

### Speckle tracking echocardiography-based strain imaging

Speckle tracking echocardiography-based strain analysis of 2D echocardiographic images acquired from the long-axis views of B-mode images (Vevo 2100, VisualSonics Inc., Toronto, Canada). All images were acquired at a frame rate exceeding 200 frames per second. The strain imaging analysis was performed using VevoStrain software (VisualSonics Inc., Toronto, Canada). Briefly, appropriate B-mode loops were selected from digitally acquired echocardiographic images based on clear visualization of the endocardial border and the absence of image artifacts. The long-axis view of the left ventricular myocardium was divided into six segments for regional speckle-tracking-based strain analysis. Global and regional strains were calculated by tracking the movement of the endocardium and epicardium border in three consecutive cardiac cycles.

### Western blot

Equal amounts (10–30 mg) of protein extracts from myocardium or cells were loaded and separated by SDS-PAGE using 8–12% acrylamide gradients. Following electrophoresis, separated proteins were transferred electrophoretically to a polyvinylidene difluoride (PVDF) membrane (Amersham Biosciences). Nonspecific proteins were blocked by incubating the membrane in a blocking buffer (5% nonfat dry milk in T-TBS containing 0.05% Tween 20) overnight. The membranes were incubated with primary antibodies against GPR22 (1:1000, Proteintech), Flag (1:1000, Sigma), Bcl-2 (Abcam 1:500), Akt (Cell signaling, 1:1000), p-Akt (Abcam, 1:3000), ERK1/2 (Cell signaling, 1:2000), p-ERK1/2 (Cell signaling, 1:1000), JNK (Cell signaling, 1:500), p-JNK (Cell signaling, 1:300), p38 (Abcam, 1:1000), p-p38 (Abcam, 1:300), and actin (Santa Cruz, 1:1000) for one hour at room temperature. Signals were detected with HRP-conjugated goat anti-mouse or goat anti-rabbit with ECL (Perkin Elmer, MA).

### Cell culture and isolation of cardiomyocytes

Eight-week-old MYH6-Flag-GPR22 male mice were used for isolating ventricular cardiomyocytes, according to a previous study [18]. For euthanasia, mice were anesthetized with 5% isoflurane inhalation, and the chest was opened to expose the heart. Following chest opening and descending aorta incision, the heart was promptly flushed with 7 mL of EDTA buffer via injection into the right ventricle. Subsequently, the ascending aorta was clamped using Reynolds forceps, and the heart was transferred to a 60-mm dish containing fresh EDTA buffer. Digestion was achieved by sequential injection of 10 mL EDTA buffer, 3 mL perfusion buffer, and 30 to 50 mL collagenase buffer into the left ventricle (LV). The myocardial tissues were then carefully dissected into 1-mm pieces using forceps. Cellular dissociation was facilitated by gentle trituration for 2 min, followed by the addition of 5 mL of stop buffer. The resulting cell suspension was filtered through a 100-µm filter, and the cells underwent four sequential rounds of gravity settling over a duration of 20 min each. The resulting cell pellet was enriched with myocytes, while the supernatant contained nonmyocyte cardiac populations. For culturing cardiomyocyte cell lines, H9C2 and RL-14 cells were purchased from ATCC and cultured in Dulbecco’s Modified Eagle’s Medium (DMEM) with 10% fetal bovine serum and DMEM/F-12 with 12.5% FBS, respectively.

### Immunofluorescent staining and histopathological examination

Animals were euthanized by heart removal following anesthetization with inhalation of 5% isoflurane. For immunofluorescent staining, isolated myocardium was embedded in optimal cutting temperature (OCT) compound and used for preparing cryosections, whereas cardiomyocytes were seeded on coverslips and cultured for 24 h. Cryosections or coverslips were fixed and permeabilized using either ice-cold acetone or 4% paraformaldehyde supplemented with 0.5% Triton X-100, followed by overnight incubation at 4 °C with antibodies against Flag and GPR22. Subsequently, coverslips or slides were incubated with Alexa Fluor 488 or Alexa Fluor 594-conjugated secondary antibodies against mouse or rabbit IgG (Invitrogen, Carlsbad, CA, USA). After counterstaining with DAPI, samples were visualized under a fluorescent microscope. To assess the extent of collagen synthesis and deposition, cardiac paraffin Sect. (6 μm thick) at 3 mm intervals were subjected to Masson’s Trichrome (MTC) staining. The staining procedure followed the manufacturer’s protocol: sections were initially fixed in Bouin’s solution, then incubated in Weigert’s Iron Hematoxylin solution, followed by staining with Biebrich Scarlet-Acid Fuchsin and Aniline Blue. Subsequently, sections were dehydrated in ethanol and xylene. For Hematoxylin and Eosin (H&E) staining, cryo-sections were fixed with 10% buffered formalin and incubated with hematoxylin for nuclear staining, while cytoplasm was stained with eosin. Sections were mounted with a mounting medium after ethanol dehydration.

### Statistical analysis

Data were expressed as mean values with standard deviation (mean ± SD). One-way ANOVA was used to evaluate the significance of differences among the groups, followed by Bonferroni multiple comparison post hoc test. Statistical analysis was performed using Prism 9.2 statistical software (GraphPad Software, La Jolla, CA, USA). A probability value < 0.05 was considered to be statistically significant.

## Results

### Chemical hypoxia reduced expression of GPR22 in cultured cardiomyocytes

**Fig. 1:**
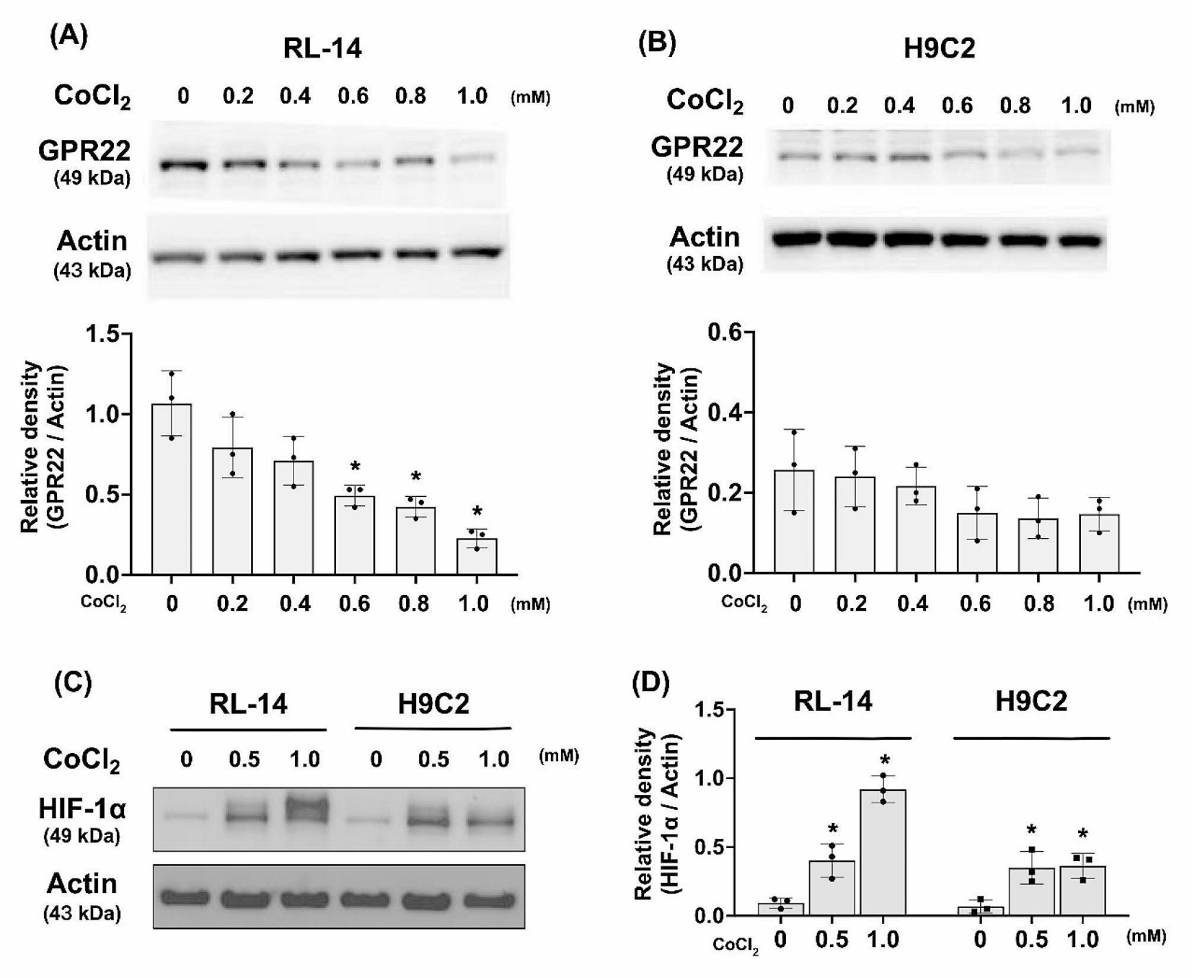
Protein expression of GPR22 in cultured cardiomyocytes under chemical hypoxia. Protein expression of GPR22 in cultured cardiomyocytes under chemical hypoxia. RL-14 (human ventricular cardiomyocytes) and H9C2 (Rat cardiac myoblasts) were incubated with CoCl_2_ (0–1 mM) for 24 h to induce chemical hypoxia. Western blots were used to determine the expression level of GPR22 and hypoxia inducible factor (HIF)-1α, respectively. (**A**) The protein expression level of GPR22 in RL-14 cells with a gradual increase in CoCl_2_. (**B**) The protein expression level of GPR22 in H9C2 cells with a gradual increase in CoCl_2_. (**C-D**) To validate cobalt chloride-stimulated hypoxia response in cells, protein expressions of HIF-1α were examined with Western blottings. Along with the increase in cobalt chloride concentration and HIF-1α expression, a gradual decrease in GPR22 expression in both RL-14 and H9C2 cells was observed. *n* = 3 for each group, * indicates a statistical significance compared with the control group (0 mM of CoCl_2_). Full-length blots are presented in Supplementary Fig. 2

To assess the impact of hypoxia on GPR22 regulation in cardiomyocytes, two cultured cardiomyocyte cell lines, RL-14 (human ventricular cardiomyocytes) and H9C2 (rat cardiac myoblasts), were subjected to chemical hypoxia induced by cobalt chloride treatment (Fig. 1). In both RL-14 and H9C2 cells, the expression levels of GPR22 exhibited a gradual decrease with increasing concentrations of cobalt chloride (Fig. 1, A-B). Furthermore, elevated expression levels of hypoxia-inducible factor (HIF)-1α were detected in H9C2 cells treated with cobalt chloride, suggesting a cellular stress response to cobalt chloride-induced chemical hypoxia (Fig. 1, C-D).

### Generation of cardiomyocyte-specific GPR22 overexpression mice

**Fig. 2:**
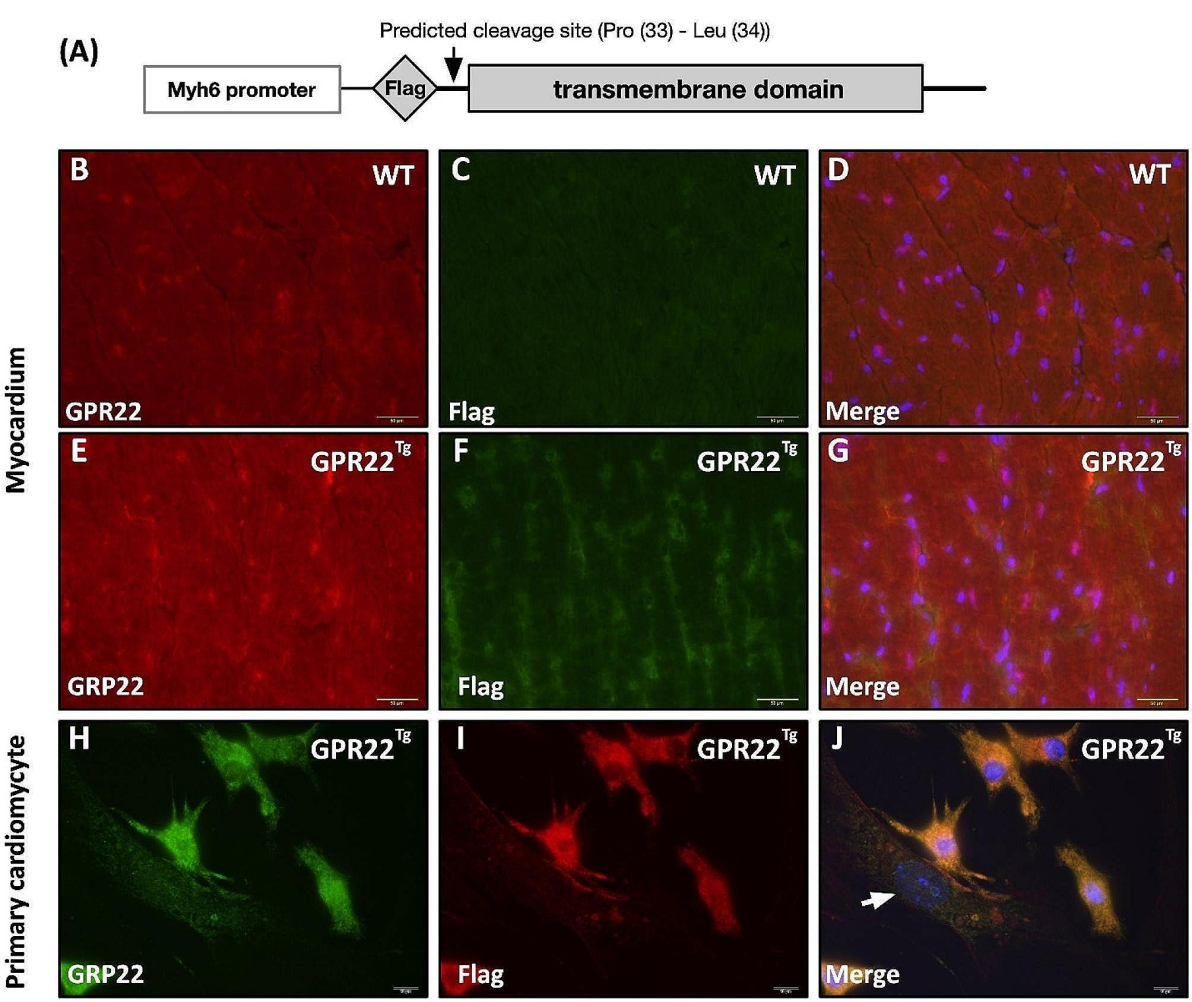
Generation of cardiomyocyte-specific GPR22 overexpression mice. (**A**) Schematic presentation of plasmid for exogenous GPR22 expression in cardiomyocytes. A predicted endogenous cleavage site was localized at amino acid position 33–34 in the N-terminal extracellular domain of GPR22. (**B-G**) Examination of exogenous and endogenous GPR22 expression in the myocardium of MYH6-Flag-GPR22 mice with immunofluorescent stainings. (**H-J**) Distribution of exogenous and endogenous GPR22 in primary adult cardiomyocytes. Expressions of Flag-tag and GPR22 were observed in cardiomyocytes but not in non-cardiomyocyte cells (white arrows). WT, wild-type mice. GPR22Tg, MYH6-Flag-GPR22 transgenic mice

To investigate the role of GPR22 expression in the myocardial stress response to ischemic conditions, we generated a transgenic mouse model with cardiomyocyte-specific overexpression of GPR22 and induced AMI via left coronary artery ligation. To achieve cardiomyocyte-specific overexpression of GPR22, we synthesized full-length murine GPR22 cDNA and inserted it into a vector containing the MYH6 promoter and a 3X Flag tag (MYH6-Flag-GPR22, Fig. 2A). Zygote injection with linearized MYH6-Flag-GPR22 plasmid was utilized to generate GPR22 transgenic mice, which were subsequently crossed with wild type mice and genotyped using PCR. Antibodies against the Flag tag and GPR22 were used to examine the distribution of endogenous and exogenous GPR22 and to assess the predicted N-terminal protein cleavage at amino acid positions 33 (Pro)–34 (Leu). Immunostaining of myocardial sections revealed distinct intracellular distribution patterns of GPR22 and the Flag tag in cardiomyocytes, suggesting N-terminal cleavage in GPR22 (Fig. 2, B-G). To further confirm the cardiomyocyte-specific expression of exogenous GPR22, adult cardiac cells were isolated from the myocardium of 8-week-old MYH6-Flag-GPR22 mice and subjected to immunofluorescent staining. The results demonstrated that immunofluorescent signals of GPR22 and the Flag tag were exclusively detected in cardiomyocytes, with no signal observed in other non-cardiomyocyte cells (Fig. 2, H-J). Interestingly, unlike the observation in myocardial sections, colocalization of GPR22 and the Flag tag was observed in isolated adult cardiomyocytes.

### Cardiac overexpression of GPR22 decreased myocardial infarcted area in mice with acute myocardial infarction

**Fig. 3:**
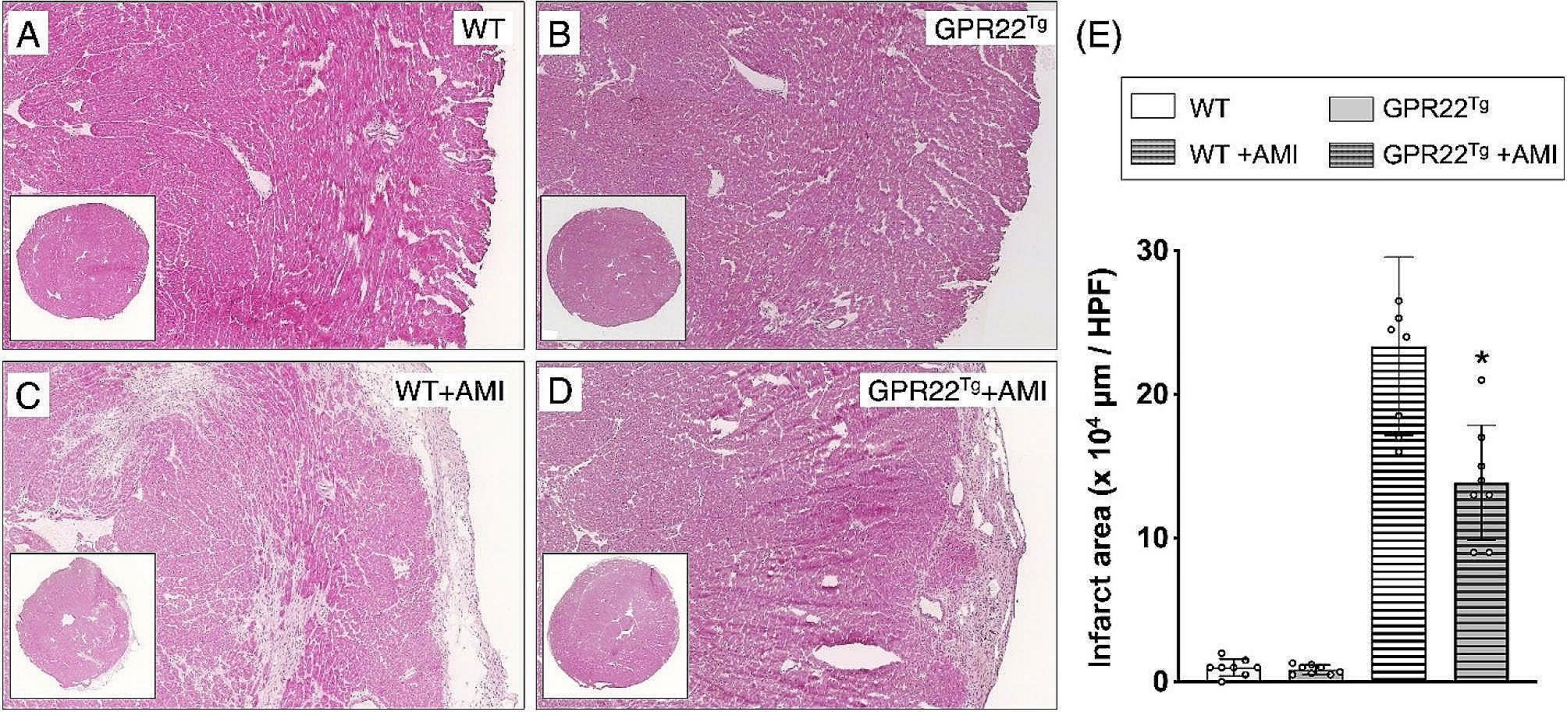
Histopathological examinations to assess the influence of GPR22 overexpression on myocardial fibrosis after acute myocardial infarction. (**A-D**) Four weeks after induction of AMI, hematoxylin and eosin (H&E) stainings were applied to myocardial cryosections. (**E**) Comparisons of myocardial infarct areas among different treatment groups (**E**). A significant increase in infarct area was observed in wild-type mice with AMI, while cardiomyocyte-specific overexpression of GPR22 reduced the myocardial infarct area. WT, wild-type mice. GPR22Tg, MYH6-Flag-GPR22 transgenic mice. *n* = 6 for each group. * indicates a statistical significance compared with the WT-AMI group

**Fig. 4:**
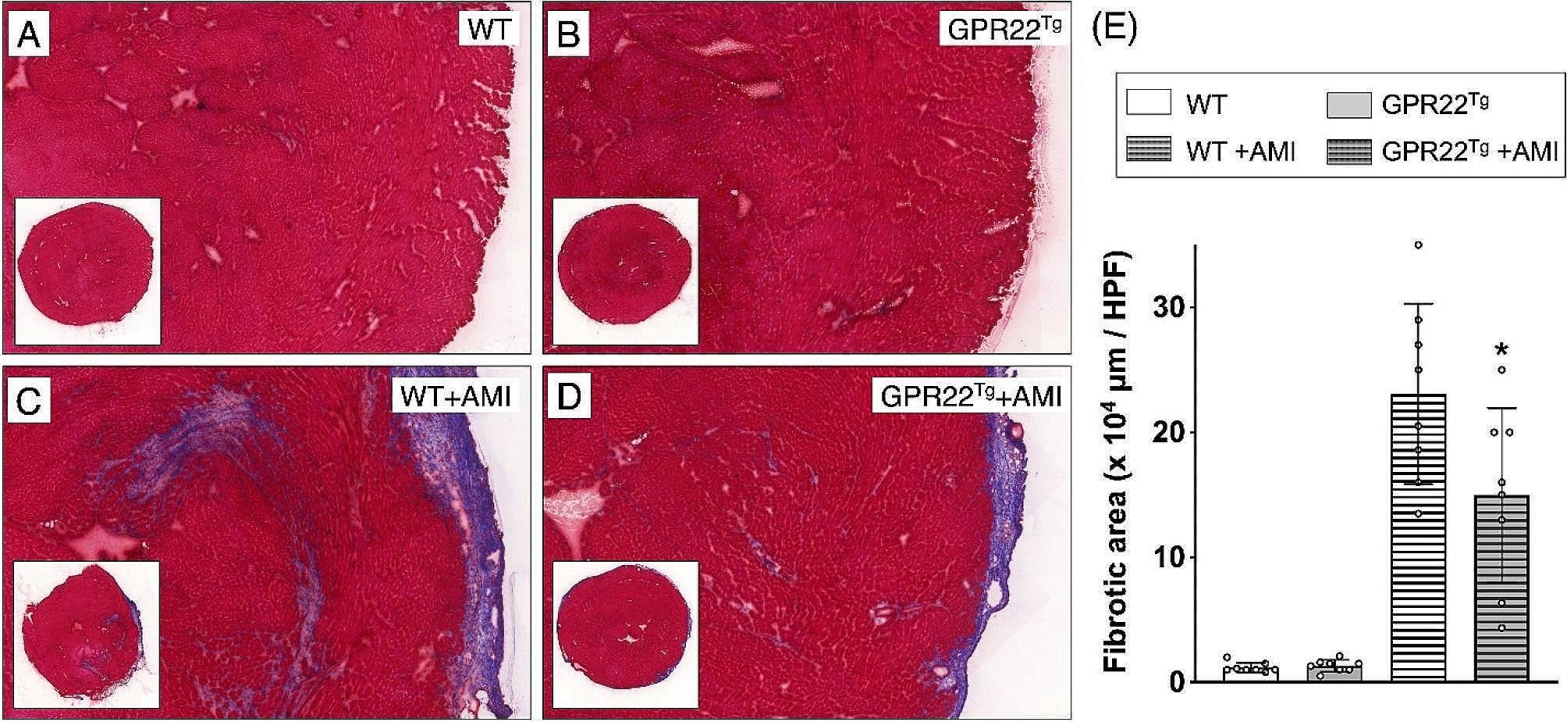
Examination of myocardial collagen deposition in mice with acute myocardial infarction. (**A-D**) Four weeks after induction of AMI, Masson’s Trichrome (MTC) stainings were applied on cryosections of the myocardium to examine collagen deposition (blue color). (**E**) Comparisons of myocardial collagen deposition areas among different treatment groups. A significant increase in myocardial collagen deposition was observed in wild-type mice with AMI, while the AMI-induced collagen deposition was reduced by overexpression of GPR22 in cardiomyocytes. WT, wild-type mice. GPR22Tg, MYH6-Flag-GPR22 transgenic mice. *n* = 6 for each group. * indicates a statistical significance compared with the WT-AMI group

Left coronary artery ligation was used to induce AMI in MYH6-Flag-GPR22 transgenic mice, aiming to investigate the impact of myocardial GPR22 overexpression on myocardial ischemic stress. Four weeks post-AMI induction, myocardial dysfunction and remodeling were assessed using echocardiographic strain images (Fig. S1). To evaluate ischemia-induced myocardial infarction and collagen deposition, two indices of cardiac remodeling, histopathological examinations with H&E staining (Fig. 3, A-D) and MTC staining (Fig. 4, A-D), were conducted on myocardial sections of mice four weeks post-AMI. H&E staining revealed an increase in infarct area in mice with AMI, whereas myocardial infarction induced by AMI was attenuated in mice overexpressing myocardial GPR22 (Fig. 3E). Similarly, compared to wild-type mice, areas of myocardial ischemia-induced collagen deposition were reduced in mice with GPR22 overexpression (Fig. 4E).

### Myocardial overexpression of GPR22 regulated the expression of apoptosis-associated proteins and activation of PI3K/Akt signaling

**Fig. 5:**
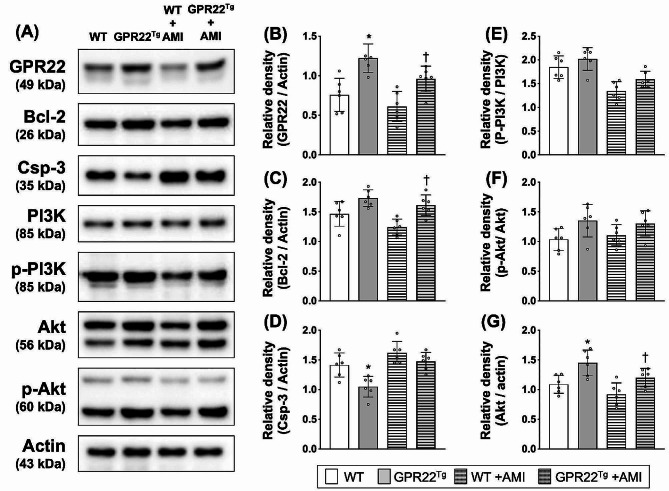
Myocardial expression of proteins involved in apoptosis and PI3K-Akt signaling. (**A**) Western blots to examine the protein expression of GPR22, Bcl-2, Caspase-3, PI3K, phosphorylated PI3K, Akt, phosphorylated Akt, and actin. (**B-G**) Comparisons of protein expression among different treatment groups. An increase in myocardial expression of GPR22 and Bcl-2 was observed in MYH6-Flag-GPR22 transgenic mice with AMI, while GPR22 overexpression reduced the level of caspase-3 in the myocardium. In both normal and AMI conditions, myocardial levels of phosphorylated Akt in MYH6-Flag-GPR22 mice were higher than those without GPR22 overexpression. WT, wild-type mice. GPR22Tg, MYH6-Flag-GPR22 transgenic mice. *n* = 6 for each group. * indicates a statistical significance compared with the WT group. † indicates a statistical significance compared with the WT-AMI group. Full-length blots are presented in Supplementary Fig. 2

Previous studies have shown that GPR22 couples constitutively to Gαi/o and leads to the inhibition of adenyl cyclase [16], whose activation increases intracellular level of cAMP and regulates cardiac contractility [20]. In addition to cAMP-dependent signaling, activation of PI3K-Akt signaling was found to be regulated by Gαi and play a role in regulating apoptosis [21]. To clarify the underlying mechanisms mediating the protective effect of GPR22 against myocardial ischemic stress, we examined the activation of PI3K-Akt signaling and the expression of apoptosis-associated proteins in the myocardium of mice four weeks after AMI induction (Fig. 5A). Compared to wild-type mice, myocardial protein expression of GPR22 was higher in MYH6-Flag-GPR22 transgenic mice. Furthermore, the expression of myocardial GPR22 was reduced with induction of AMI, while GPR22 transgene replenished its expression level in the myocardium (Fig. 5B). In mice with AMI, myocardial expression of Bcl-2, an anti-apoptotic protein, was higher in GPR22 overexpression mice compared to wild-type mice (Fig. 5C). Additionally, a significant decrease in pro-apoptotic caspase-3 expression was observed in GPR22 overexpression mice under normal condition (Fig. 5D). Although the ratios of phosphorylated PI3K to total PI3K and phosphorylated Akt to total Akt showed no statistical difference between mice with or without GPR22 overexpression (Fig. 5, E-F), myocardial levels of phosphorylated Akt in MYH6-Flag-GPR22 mice were higher than those without GPR22 overexpression (Fig. 5, E-G).

### Myocardial overexpression of GPR22 downregulated activation of ERK signaling in mice with acute myocardial infarction

**Fig. 6:**
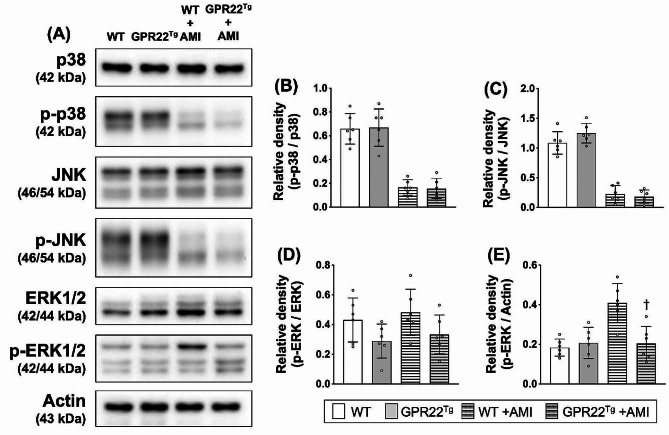
Myocardial expression and activation of MAPK signaling proteins. (**A**) Western blottings were used to examine the protein levels of total p38, phosphorylated p38, total JNK, phosphorylated JNK, total ERK1/2, phosphorylated ERK1/2, and actin. (**B-E**) Comparisons of protein expressions among different treatment groups. In both normal and AMI conditions, ratios of phosphorylated p38 to total p38 and phosphorylated JNK to total JNK showed no statistical significance between WT mice and MYH6-Flag-GPR22 mice (**B-D**). In AMI condition, myocardial levels of phosphorylated ERK1/2 in MYH6-Flag-GPR22 mice were lower than that without GPR22 overexpression. WT, wild-type mice. GPR22Tg, MYH6-Flag-GPR22 transgenic mice. *n* = 6 for each group. * indicates a statistical significance compared with the WT group. † indicates a statistical significance compared with the WT-AMI group. Full-length blots are presented in Supplementary Fig. 2

In addition to PI3K-Akt signaling, we also investigated the myocardial expression and activation of MAPK signal transduction proteins, which are known to be regulated by GPCR signaling and involved in the cardiac stress response (Fig. 6A). Although the activation of MAPK-p38 and JNK was reduced following AMI induction, the ratios of phosphorylated p38 to total p38 and phosphorylated JNK to total JNK did not differ between wild-type and GPR22 overexpression mice (Fig. 6, B-C). However, while the ratio of phosphorylated ERK1/2 to total ERK1/2 showed no statistical significance between WT mice and AMI mice (Fig. 6D), myocardial levels of phosphorylated ERK1/2 in MYH6-Flag-GPR22 mice post-AMI were lower than those without GPR22 overexpression (Fig. 6E).

## Discussion

Myocardial infarction (MI), one of the most life-threatening coronary events, is a leading cause of global morbidity and mortality and the primary contributor to heart failure [22]. Recent meta-analysis studies indicated a global 9.5% prevalence of MI among individuals older than 60 years [23]. The limited regenerative capacity of the cardiomyocytes following MI results in cardiac remodeling, a significant cellular interstitial change in the myocardium [24]. During cardiac remodeling, inflammation, oxidative stress, activated cardiac fibroblast, and disordered extracellular matrix collectively contribute to myocardial fibrosis and cardiac dysfunction [25]. Moreover, the recruitment of immune cells, and intercellular communication among cardiomyocytes, cardiac fibroblasts, and immune cells have been identified to play crucial roles in the pathophysiology of MI [26–29]. A recent study further indicated that cutaneous lupus erythematosus, an autoimmune skin disease, is associated with a higher risk of arterial diseases and MI [30]. Beyond its impact on cardiac function, neuropsychiatric disorders following circulatory disorders are significantly more common and result in higher mortality rates post-MI compared to individuals without MI [31]. The development of novel diagnostic techniques and therapeutic strategies through preclinical studies and clinical validation has been a crucial focus in cardiovascular research [32–34].

Expressions of over 200 GPCRs have been observed in hearts [35]. Although ligands and downstream signalings of certain myocardial GPCRs have been revealed in past decades [9, 20], the physiological roles and downstream effectors of numerous myocardial orphan GPCRs, including GPR22, remain largely unknown. GPR22 was originally identified as a neuronal GPCR expressed in several specific brain regions, including the frontal cortex, caudate, and thalamus [36]. Recent studies also indicated a decrease in GPR22 expression in the brain of Alzheimer’s disease (AD) patients [14, 37]. Concurrently, reduced expression of GPR22 was observed in the myocardium of rodents with ventricular pressure overload and diabetes [15, 16]. Our recent study further demonstrated that maternal nutritional insult reduces myocardial GPR22 expression and leads to exacerbated cardiac remodeling in offspring with ventricular pressure overload [18]. These findings suggest a negative correlation between GPR22 expression and various types of cardiac stresses. In addition to mechanical and nutrition stress, our present study indicates that the expression of GPR22 is downregulated by hypoxic and ischemic stress. Insights from clinical observations in AD and studies in animal models exposed to different types of cardiac stresses collectively suggest that decreased GPR22 expression could serve as a stress indicator or an index for predicting organ failure, particularly in the brain and heart.

So far, the ligand or activator of GPR22 remains unknown, while its intracellular trafficking and downstream signaling are also not clear. Previous studies have demonstrated that N-terminal cleavage in GPCRs, such as that in β1-adrenergic receptors and proteinase-activated receptors (PARs), is associated with the activation, translocation, and stabilization of GPCRs [38–41]. In this study, we utilized an N-terminal Flag-tag to label full-length GPR22 for monitoring the distribution, translocation, and protein cleavage of GPR22. In the myocardial section of MYH6-Flag-GPR22 transgenic mice, distinct distribution patterns of GPR22 and Flag-tag were observed, indicating the existence of N-terminal cleavage of GPR22 under normal physiological conditions (Fig. 2, B-G). Of interest, in isolated adult cardiomyocytes, whose intercellular interaction as well as microenvironments were disrupted, a cytoplasmic co-localization of GPR22 and Flag-tag was found (Fig. 2, H-J). This result suggests that the N-terminal cleavage and membrane translocation of GPR22 were blocked in cardiomyocytes under physiological or environmental stresses. While the N-terminal cleavage of GPR22 may be associated with its stability, degradation, and downstream signaling activation during cellular stress responses remains unclear.

A previous study using mice with GPR22 depletion has revealed the necessity of GPR22 in preserving cardiac function after ventricular pressure overload [16]. Although GPR22 was found to couple with Gαi/o and reduce the intracellular level of cAMP [16], the detailed underlying mechanisms linked to cardiac stress response and prognosis remain unclear. Among GPCR downstream signaling pathways, PI3K-Akt and MAPK signalings are considered to participate in regulating survival and apoptosis of cardiomyocytes under myocardial ischemia [42–44]. Through examinations on expression and phosphorylation levels of PI3K-Akt and MAPK (MAPK-p38, JNK, ERK1/2) signaling proteins, an upregulation of PI3K-Akt signaling as well as a downregulation of ERK1/2 phosphorylation were observed in the myocardium of with GPR22 overexpression and AMI. This finding echoes a previous study in which the hypoxia-induced apoptosis in neonatal cardiomyocytes was protected by activation of PI3K-Akt signaling through β2-adrenergic receptor-coupled Gαi/o [21].

Despite the widespread use of G protein-coupled receptor (GPCR)-targeted drugs in cardiovascular diseases, such as those targeting β-Adrenergic receptors (βARs), angiotensin II type 1 receptors (AT1Rs), and glucagon-like peptide-1 (GLP-1) receptors [32, 45], only specific types of GPCRs, including β2-adrenergic receptors, opioid receptors, and adenosine receptors, have been identified to exert cardiomyocyte protective functions against ischemic stress [21, 46, 47]. In our previous study, we have demonstrated a cardiomyocyte-specific expression of GPR22 in the myocardium [18]. The present study, utilizing a transgenic mouse model, further indicated an increased expression of GPR22 in cardiomyocytes and showed that upregulating its downstream signaling pathways could ameliorate myocardial injury following acute myocardial infarction (AMI). These findings suggest a potential clinical application of GPR22 in the management of ischemic heart diseases. However, there are limitations to the present study. We only used a gain-of-function model to evaluate the role of GPR22 in AMI. Future studies incorporating additional cell models with GPR22 silencing may provide further evidence to validate the physiological role of GPR22 in cardiomyocytes.

## Conclusion

In this study, through induction of AMI on a mouse model with cardiomyocyte-specific GPR22 overexpression mice, we demonstrated that ischemic stress reduces the expression of GPR22 in the myocardium. Overexpression of GPR22 in cardiomyocytes ameliorates myocardial infarction and collagen deposition in mice post-AMI through regulating myocardial activation of PI3K-Akt signaling.

### Electronic supplementary material

Below is the link to the electronic supplementary material.

Supplementary Material 1

Supplementary Material 2

## Data Availability

The datasets during and/or analysed during the current study available from the corresponding author on reasonable request.
