# Supplementary material for: Cardiomyocyte-specific overexpression of GPR22 ameliorates cardiac injury in mice with acute myocardial infarction
Source: BMC Cardiovasc Disord. 2024 May 30;24:287. doi: 10.1186/s12872-024-03953-5 (PMC11138089; doi:10.1186/s12872-024-03953-5)
Supplement: Supplementary file 1 — Supplementary Material 1 [file 12872_2024_3953_MOESM1_ESM.docx]

**Supplemental Figure 1**

1. GPR22 transgenic mouse


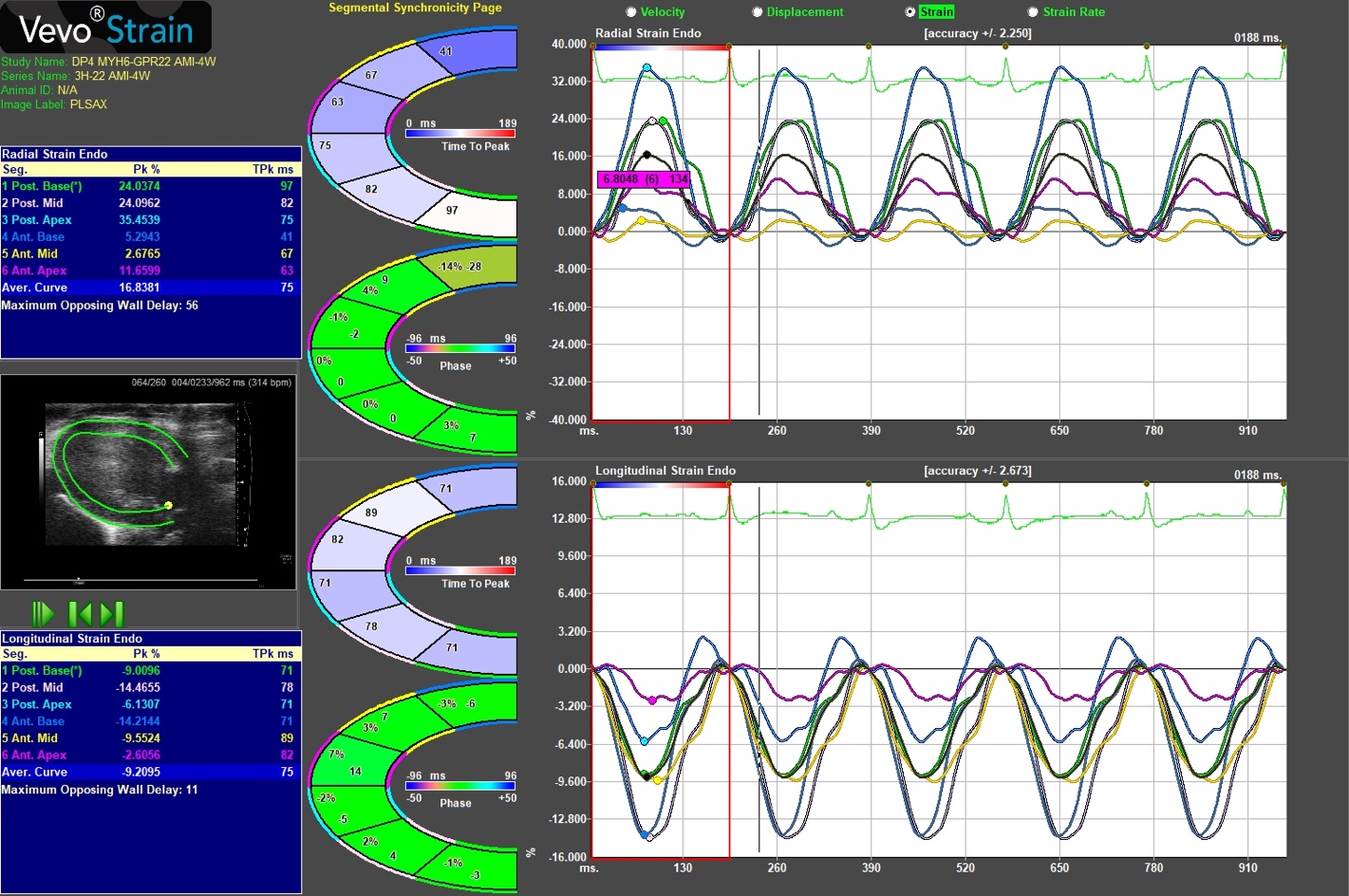


1. GPR22 transgenic mouse 4 weeks post-MI


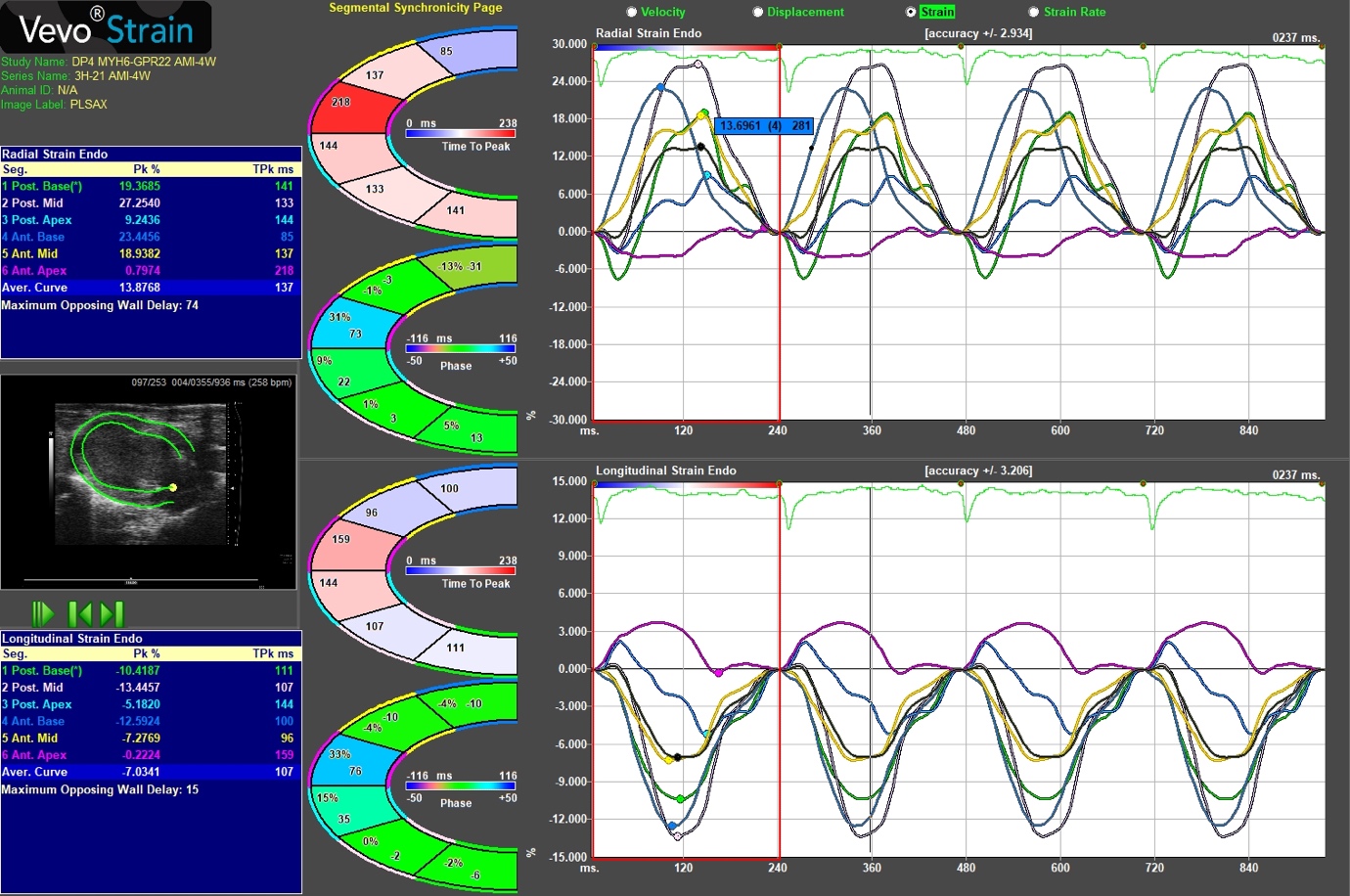


1. Wild type mouse 4 weeks post-MI


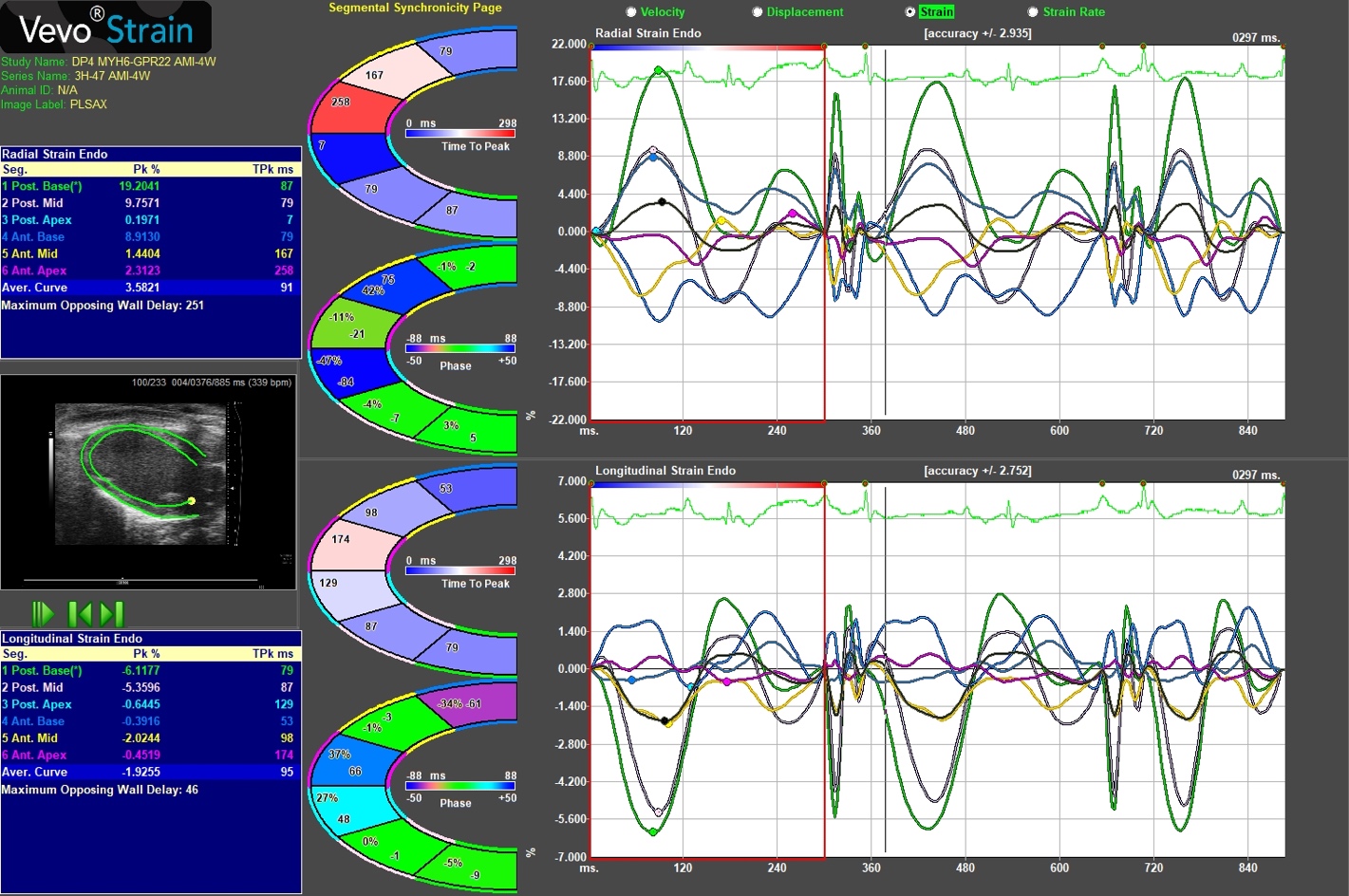


**Evaluation of myocardial infarction with echocardiographic strain image.**

Four weeks after induction of acute myocardial infarction (AMI), echocardiographic strain image analysis was performed to determine cardiac function and remodeling (Vevo 2100 imaging system, Visual Sonics).(A) Echocardiographic strain image of a GPR22 transgenic mouse without induction of AMI. (B) Echocardiographic strain image of a GPR22 transgenic mouse 4 weeks post-AMI. (C) Echocardiographic strain image of a wild type mouse 4 weeks post-AMI. Ventricular dyssynchrony as well as increased Time to Peak (TPK) at left coronary artery-supplied segemnts (anterior-apex and anterior-mid segments) were observed in mice with AMI.
