# Supplementary figures and images for: Cardiomyocyte-specific overexpression of GPR22 ameliorates cardiac injury in mice with acute myocardial infarction
Source: BMC Cardiovasc Disord. 2024 May 30;24:287. doi: 10.1186/s12872-024-03953-5 (PMC11138089; doi:10.1186/s12872-024-03953-5)

Fig. 1A

RL-14

GPR22

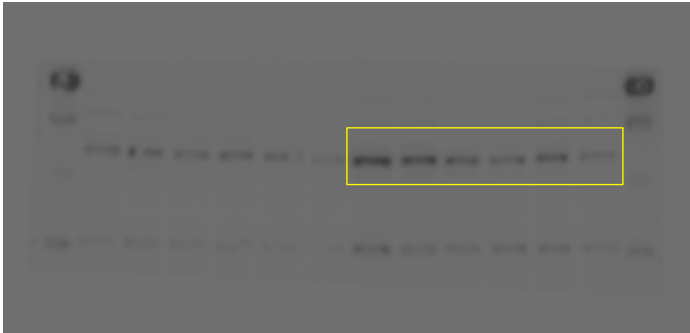

Actin

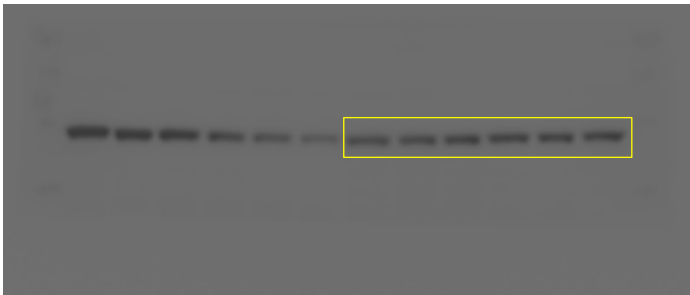

Fig. 1B

H9C2

GPR22

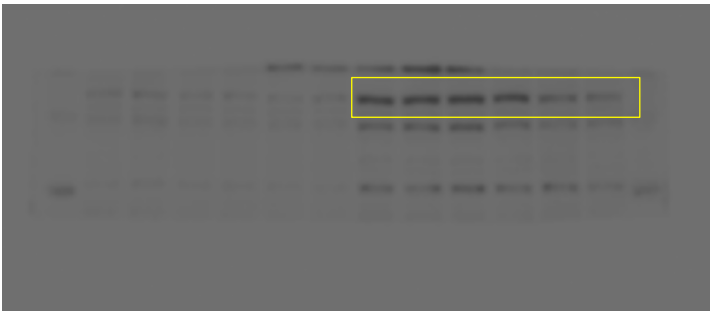

Actin

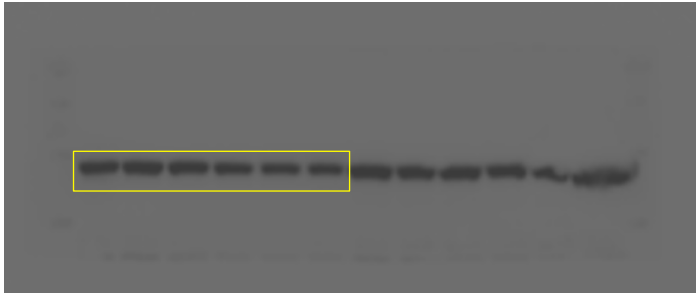

Fig. 1C

RL-14

H9C2

HIF-1 $\alpha$

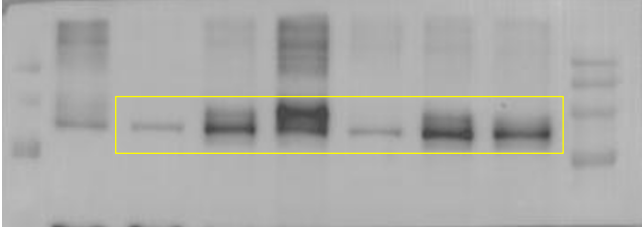

Actin

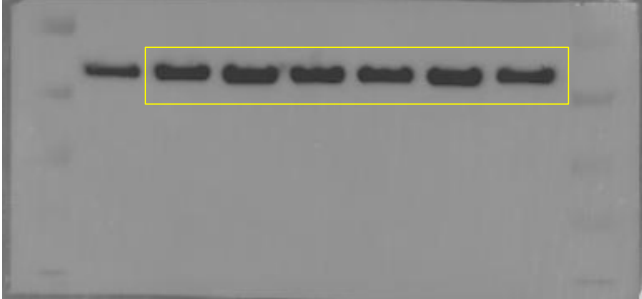

Fig. 5A

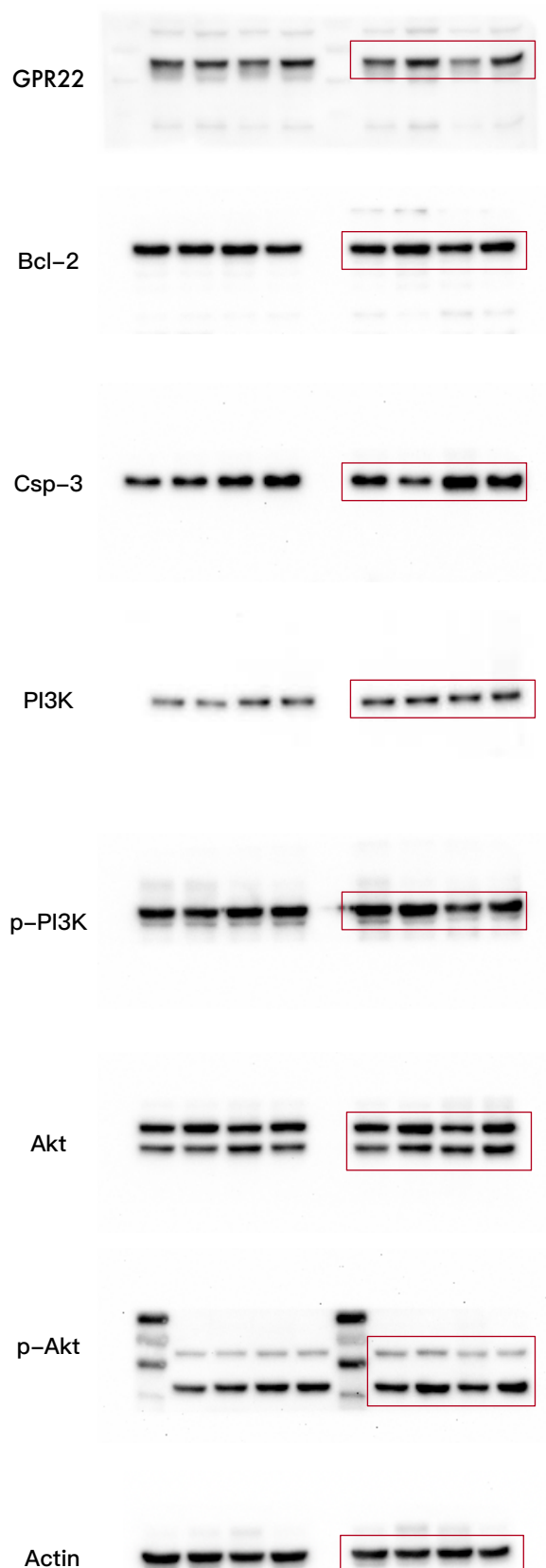

Fig. 6A

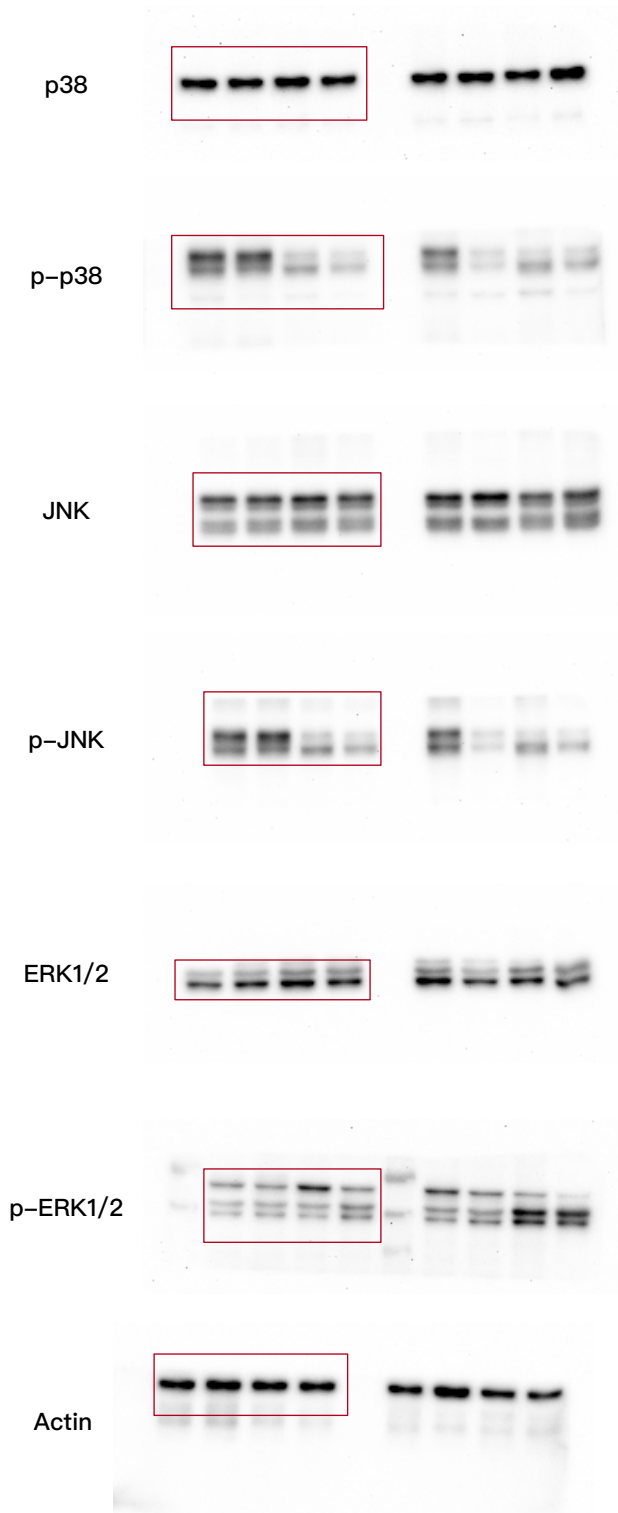

Supplement: Supplementary file 2 — Supplementary Material 2 [file 12872_2024_3953_MOESM2_ESM.pdf]
